# Microbiota Is Involved in Post-resection Adaptation in Humans with Short Bowel Syndrome

**DOI:** 10.3389/fphys.2017.00224

**Published:** 2017-04-19

**Authors:** Laura Gillard, Camille Mayeur, Véronique Robert, Isabelle Pingenot, Johanne Le Beyec, André Bado, Patricia Lepage, Muriel Thomas, Francisca Joly

**Affiliations:** ^1^Institut National de la Santé et de la Recherche Médicale UMR 1149, L'Unité de Formation de Recherche de Médecine Paris Diderot, Université Paris Diderot, Sorbonne Paris Cité, Assistance Publique – Hôpitaux de Paris, Départements Hospitalo Universitaires UnityParis, France; ^2^Micalis Institute, Institut National de la Recherche Agronomique, AgroParisTech, Université Paris-SaclayJouy-en-Josas, France; ^3^Service de Gastroenterologie et Assistance Nutritive, Hôpital BeaujonClichy, France; ^4^Assistance Publique – Hôpitaux de Paris, Hôpital Pitié-Salpêtrière-Charles Foix, Biochimie Endocrinienne et OncologiqueParis, France; ^5^Université Pierre et Marie Curie, Sorbonne UniversitéParis, France

**Keywords:** entero-endocrine hormones, resection, dysbiosis, lactate, microbiome, Glucagon-like peptide, short chain fatty acids

## Abstract

Short bowel syndrome (SBS) is characterized by severe intestinal malabsorption following restrictive surgery. The objective of this study was to determine the functional contribution of SBS-microbiota after resection. It is well-known that SBS-microbiota displayed specific features with a prevalence of Lactobacillus, a low amount of some anaerobic microbes (*Clostridium leptum*) and an accumulation of fecal lactate in some patients. Patients with jejuno-colonic anastomosis were stratified according to the presence of lactate in their feces and, we observe that the lactate-producing bacteria were predominant in the sub-group of patients accumulating fecal lactate. One case of D-encephalopathy crisis occurred when the D-lactate isoform accumulated in the feces and plasma bicarbonate levels decreased. The fecal sample at the time of the encephalopathy was transferred to germ free rats (SBS-H rats). The SBS-H microbiota conserved some characteristics of the SBS donnor, predominated by lactate-producing bacteria (mainly *Lactobacillus*), a low level of lactate-consuming bacteria and undetectable *C. leptum*. However, lactate did not accumulate in feces of recipient rats and the D-encephalopathy was not reproduced in SBS-H rats. This suggests that the intact small bowel of the recipient rats protected them from lactate accumulation and that D-lactate encephalopathy can occur only in the absence of small intestine. After fecal transfer, we also show that gnotobiotic rats exhibited high levels of circulating GLP-1 and ghrelin, two hormones that are known to be induced in SBS patients. Therefore, the microbiota of SBS is a reservoir of biological signals involved in post-resection adaptation.

## Introduction

Short bowel syndrome (SBS) results from extensive surgical resection leaving a small bowel remnant length of <200 cm (Pironi et al., 2015). SBS is a rare disease, with a prevalence of 1.4 cases per million people in Europe. The large reduction of gut absorptive area results in the absorption of macronutrients, water, and electrolytes that is below the minimum necessary to meet the nutritional needs of the patient. As a consequence, patients require parenteral nutrition (PN). The severity of SBS is related to its etiology, the length of resection, and the degree of adaptation of the remnant bowel (Pironi et al., 2015; Mayeur et al., 2016). Some adaptive processes occur during the first 2 years after the surgery that improve the absorption of hydro-electrolytes and energy with a decrease of diarrhea (Nordgaard et al., 1996). PN may be stopped in SBS patients when the adaptive process compensates for the malabsorption (Jeppesen, 2014). In SBS patients with a colon in continuity, i.e., following jejuno-colonic or jejuno-ileal anastomosis, the remnant colon displays morphological, endocrine, and microbiological adaptations: (i) an increase in the absorptive area, with a higher number of cells and deeper crypts (Joly et al., 2009), (ii) an increase in Glucagon-like peptide GLP-1, GLP-2 and plasma ghrelin levels (Jeppesen et al., 2000, 2001; Gillard et al., 2016) and finally, (iii) modification of the fecal microbiota of SBS patients, characterized by the high prevalence of *Lactobacillus*, sub-dominance of *Bacteroidetes*, major depletion of *Clostridium coccoides*, and the absence of *Clostridium leptum* (Joly et al., 2010). Concomitant with these local intestinal adaptations, 70% of adult SBS patients develop hyperphagia and spontaneously increase their food intake (Messing et al., 1991; Crenn et al., 2004). The administration of GLP-1 and GLP-2 hormones (or analogs) is efficient to enhance the natural adaptation process and reduce PN (Drucker et al., 1996; Jeppesen, 2012; Madsen et al., 2013). Thus, the adaptation following intestinal resection includes spontaneous, complex intestinal, and systemic compensatory processes that could be improved by therapeutic treatments.

The intestinal microbiota plays a key role in energy salvage and digestive functions (Cherbuy et al., 2010; Tomas et al., 2015; Verbeke et al., 2015; Deschemin et al., 2016); but the SBS-microbiota is especially efficient in energy recovery as it provides more energy (up to 1000 Kcal) than the microbiota of healthy humans (up to 200 Kcal; McNeil, 1984; Nordgaard et al., 1994, 1996; Briet et al., 1995). The SBS-microbiota, since rich in Lactobacillus, leads to the accumulation of fecal lactate in some patients.

Lactate does not accumulate in healthy human feces because it is absorbed by intestinal cells or used by lactate consuming bacteria. In some SBS patients, the high amount of lactate found in feces indicates that production exceeds absorption capacities by host or by lactate consuming bacteria. The L- and D-forms of lactate can be produced by micro-organisms. L-lactate is rapidly metabolized and its accumulation seems not to be linked to specific disorders. In contrast, D-lactate enantiomer is neurotoxic even if the mechanisms underlying its toxicity are not well-understood. Some SBS patients develop severe, symptomatic D-lactic acidosis, with metabolic acidosis and neurological disorders (Kowlgi and Chhabra, 2015). The D-acidosis is observed only in some patients with a large small bowel resection with a part of the colon in continuity and some cases are also described in patients with bypass (Narula et al., 2000). The impact of this complication is important because unknown by clinicians and without specific preventive and curative therapy. The clinical presentation is often characterized by episodes of unusual comportment, altered mental functions, weakness, and/or impaired motor coordination. Hostile and aggressive behaviors have been also described. The correction of neurological symptoms by fasting is an additional diagnostic element. These neuropsychiatric disorders are associated with severe metabolic acidosis. The occurrence of D-lactic acidosis remains sporadic and non-predictable in SBS patients and is often difficult to diagnose since measurement of serum D-lactate concentration is not routinely done in hospitals. We observe that the D/L lactate ratio in feces is a relevant index for D-encephalopathy risk and we encourage monitoring of the D/L-fecal lactates, when patients are suspected to be at risk (Mayeur et al., 2013).

The biological signals arising from the SBS-microbiota need to be better understood as they are both beneficial (with a high ability to recover energy) and deleterious (with a potential to overproduce D-lactate). Here, we describe the gut microbial composition and fermentative activity of SBS patients classified either as lactate accumulator (LA) or non-lactate accumulator (NLA). The gut microbial composition and fermentative activity were also followed for 1 year in a patient known to develop severe recurrent and non-predictable D-encephalopathy crises. We tested the contribution of the microbiota to the physio-pathological characteristics of SBS by performing bulk fecal transfer from a patient at the time of D-encephalopathy into recipient germ-free rats. We thus highlight how gut remodeling (due to a surgery) can affect the microbiota that in turn contributes to the clinical outcome, i.e., susceptibility to D-acidosis and post-resection adaptation.

## Materials and methods

### Selection and clinical characteristics of patients with short bowel syndrome

#### Ethics statement and informed consent

The Human Investigations Committee of the Saint-Louis Hospital in Paris approved the study (no. 031048,) in January 2004. All patients gave their written informed consent to participate in this study. Inclusion took place between January 2006 and March 2013. Patients were monitored in the nutrition support unit, an approved center for intestinal failure and home parenteral nutrition, located at the Lariboisiere and Beaujon Hospitals (Paris and Clichy, respectively). Inclusion and exclusion criteria of the SBS patients were similar to those published in Joly et al. (2009). Finally, 17 patients were included (S1–S17) and the last one, with severe recurrent D-lactic encephalopathy crises, was followed and provided samples during 1 year (S17).

#### Clinical characteristics of patients and GLP-1 dosages

The clinical and nutritional data from the LA and NLA groups (Table 1) were collected as described in Mayeur et al. (2013).

**Table 1:** **Clinical characteristics and food intake of patients**.

**SBS patient**		**Sex (n)**	**Age at study (y)**	**Time to reestablishment of continuity (y)**	**Remnant small bowel length (cm)**	**Remnant colon (%)**	**BMI (kg/m^2^)**	**Bicarbonate (mmol/l)**	**Oral intake (Kcal/day)**	**Proteins (g)**	**Fats (g)**	**Carbohydrates (g)**	**Fiber (g)**	**HPN (%)**
NLA (*n* = 7)		F (*n* = 3), M (*n* = 4)	64 ± 8	13 ± 6	42 ± 21	65 ± 15	21 ± 3	23 ± 4	2, 412 ± 659	91 ± 23	95 ± 22	252 ± 79	14 ± 4	40 ± 24
LA (*n* = 9)		F (*n* = 4), M (*n* = 5)	50 ± 14	8 ± 7	50 ± 33	49 ± 17	21 ± 2	17 ± 2	2, 856 ± 921	103 ± 39	114 ± 40	296 ± 83	14 ± 3	35 ± 27
S17	Over 1 year	M (*n* = 1)	21	22	35	75	17.5 ± 0.4	27 ± 2	3,199	122	133	315	–	0
	Acidosis crisis		22				17	22	–	–	–	–	–	0

*Sixteen patients with SBS were divided into two groups (LA, Lactate accumulator; NLA, non-lactate accumulator), depending on the presence (LA, n = 9) or absence (NLA, n = 7) of lactate in the feces at the time of the study. One patient (S17) was followed for 1 year until having an acidosis crisis. The age at the date of sampling and the time since the reestablishment of continuity are expressed in years (y). The length of remaining colon in continuity is expressed as the percentage of the usual length. BMI, body mass index (kg/m^2^). Fasting plasma bicarbonate levels are expressed as mmol/L. Total oral intake is expressed in kcal/day. The repartition of proteins, fat, carbohydrates, and fiber are expressed as the grams of total daily oral intake (g/day). HPN, supply by home parenteral nutrition infusions expressed as the percentage of total intake per week. Values are expressed as the mean ± SD. F, female; M, male*.

Human GLP-1 was measured in fasting blood samples containing DPPIV inhibitor (DPPIV-010, Millipore) by ELISA (EDITM Total GLP-1 ELISA kit, KT 876, Epitope diagnostics, France). Samples were centrifuged at 3000 rpm for 10 min. Aliquoted plasma was stored at −80°C until analysis.

### Establishment of SBS-patient-gut-microbiota-humanized rats

#### Approval and accordance

All procedures were carried out according to European guidelines for the care and use of laboratory animals with permission from the French Veterinary Services dedicated to M. T (DAP 14_37).

#### Animals and study design

All germ free (GF, males, Fisher 344) animals were born and bred at the Institut National de la Recherche Agronomique (Anaxem, INRA, Jouy-en-Josas, France). To establish the humanized-SBS rats (SBS-H group, *n* = 6), we performed one oral gavage in recipient GF rats with 1 ml of S17 fecal sample freshly diluted 2.5-fold in reduced phosphate buffered saline (PBS) in an anaerobic chamber. GF control rats (*n* = 4) were mock inoculated with sterile reduced PBS. GF and SBS-H rats were 5 weeks old when inoculated by gavage. Conventional (CV, *n* = 6) male Fisher 344 rats (Charles Rivers), were reared in a specific pathogen free (SPF) facility. All animals were fed *ad libitum* with a standard diet (R03; SAFE, Augy, France) sterilized by irradiation. To challenge the *in vivo* SBS fecal microbiota functional activity, CV, and SBS-H rats received 45 g/L lactose in water *ad libitum* for the last 5 days before sacrifice. Fecal samples from SBS-H rats were collected daily for the first 3 days and twice a week for 30 days after gavage. Fecal samples (200 mg fractions) were frozen at −80°C for future analyses. All rats were 9 weeks old when sacrificed.

### Composition of fecal microbiota from SBS patients and H-SBS, GF, and CV rats

#### DNA extraction from feces

Total DNA was extracted from aliquots of 200–250 mg of fecal samples as described previously (Mayeur et al., 2013). DNA extracts were stored at −20°C for real-time quantitative PCR (qPCR) analysis of the 16S ribosomal genes and 454 pyrosequencing.

#### Evaluation of total bacterial counts by real-time qPCR analysis of bacterial 16s rRNA gene

The total load of bacteria and *C*. *leptum* present in the microbiota of SBS patients and in each group of rats was evaluated by qPCR analyses targeting “all bacteria” and the “*C. leptum* group”16S rRNA genes. We used universal primers (F-bact1369 and R-prok1492), a P-TM1389F probe for “all bacteria” and (F-Clept09 and R-Clept08) a P-Clep 01probe for the *C. leptum* group. The qPCR amplification as previously described (Mayeur et al., 2013). PCR inhibition was tested with fecal DNA dilutions using the TaqMan exogenous internal positive control (Applied Biosytems, Carlsbad, CA, USA). No inhibition was detected using 10^−3^ dilutions of fecal DNA; consequently, this dilution was used for all PCR amplifications.

#### Evaluation of microbiota composition by 454 pyrosequencing

Microbiota composition and diversity were analyzed using 454 pyrosequencing targeting the V3–V4 region of the 16S rRNA gene (V3fwd: 5′ TACGGRAGGCAGCAG 3′, V4rev: 5′ GGACTACCAGGGTATCTAAT 3′), as previously described (Le Roy et al., 2013). DNA samples were sequenced at Genoscreen (Genoscreen, Lille, France) using GS-FLX-Titanium technology following the manufacturer's instructions (Roche). Briefly, sequences were trimmed for adaptor and PCR primer removal and binned for a minimal sequence length of 300 bases. The minimal base quality threshold was set at 27 and 15% of tolerated N. Using QIIME, sequences were further clustered in Operational Taxonomic Units (OTUs) at 97% identity using Cd-hit (Li and Godzik, 2006; Caporaso et al., 2010). Representative OTUs were assigned to different taxonomic levels (from phylum to genus) and closest relative bacterial species using SEQMATCH and an up-to-date 16S rRNA gene RDP database. Estimates of OTU diversity were calculated according to the Shannon and Simpson indices.

#### Fermentative activity analyses of fecal microbiota from SBS patients and H-SBS, GF, and CV rats

The concentrations of short-chain fatty acids (acetate, propionate, and butyrate) in the fecal content were analyzed after water extraction of acidified samples using gas-liquid chromatography (Nelson 1020; Perkin-Elmer, St. Quentin en Yvelines, France), as described previously (Lan et al., 2008). Fecal SCFA concentrations are expressed as millimolar (mM). The concentration of D and L-Lactates was measured in fecal samples using the Biosentec D/L lactic acid enzymatic kit according to the manufacturer's instructions (Biosentec, Toulouse, France) and as we described previously (Rul et al., 2011). Fecal D and L-Lactate concentrations are expressed as millimolar (mM).

#### Blood collection and analysis

The day of sacrifice (30 days after colonization), all animals were fasted overnight but allowed *ad libitum* access to water. Blood was collected from the tail in chilled Heparine-coated (IU) tubes containing 1/1,000 DPP-4 inhibitor (DPP-IV-010 Millipore Millipore, France). Total rat ghrelin was measured by RIA (MI-GHRT-89HK Merck Millipore, Saint-Quentin en Yvelines, France). The determination of the plasma leptin and GLP-1 concentration was performed in duplicate for each rat using the Milliplex map rat metabolic hormones magnetic bead panel (RMHMAG-84K) according to the manufacturer's instructions. Results are expressed in pg/mL. The total GLP-1 concentration of SBS patients was determined by ELISA (Epitope Diagnostics, Eurobio, France) in the department of Endocrine and Oncologic Biochemistry at the Pitié-Salpetrière hospital (Paris).

#### Rat tissue collection and preservation

The day of sacrifice, the colon was removed and rinsed twice in PBS. A segment was used for histological analyses. The remaining part of the colon was opened and the mucosa was scrapped off and immediately frozen in liquid nitrogen and stored at –80°C until mRNA preparation.

#### Histology and immunochemistry analyses

Paraffin-embedded 4% PAF-fixed rat colons were sectioned at 5 μm. The sections were stained with Hematoxylin Phloxine Saffron and an average of 12 crypt depths was measured per animal. For immunohistochemistry, the sections were immuno-labeled with mouse monoclonal anti-GLP-1 antibody ([8G9] Abcam) and rat monoclonal anti-Ki67 antibody (DAKO, Les Ulis, France) using a detection kit (Bond Polymer Refine detection; DS9800; Leica Microsystems). The primary antibody was substituted by PBS as a negative control. The number of GLP-1-positive cells was determined by counting positive cells per μm^2^ (one labeled slide per animal) of mucosa and the number of Ki67-positive cells was determined by counting the positive cells per crypt (10 per animal) using Calopix image analysis software (TRIBVN, Chatillon, France). The results are expressed as the number of GLP-1-positive epithelial cells per μm^2^ and as the percentage of Ki67-positive epithelial cells per crypt.

#### RNA isolation and real-time PCR

Total RNA was extracted from frozen colon mucosa scrapings using Trizol reagent (Life Technologies, Saint Autin, France). The RNA yield was quantified by spectrophotometry, and the quality determined by Agilent 2100 Bioanalyzer analysis using the RNA 6000 nanoassay kit (Agilent Technologies, Santa Clara, CA, USA). The obtained RNA integrity number (RIN) indicated good RNA quality for all samples (average RIN of 8). Reverse transcription was performed with 8 μg of total RNA using a high-capacity cDNA reverse transcription kit (Applied Biosytems) following the manufacturer's instructions. Real-time qPCR was performed using the ABI PRISM 7000 sequence detection system and TaqMan universal PCR technology. Probes were from Applied Biosytems: *SGLT-1* (Rn01640634_m1), *PepT-1* (Rn01466071_m1), *SMCT-1* (Rn01503812-m1), *MCT-1* (Rn00562332-m1), *Nhe3* (Rn00709709-m1), *Aquaporin-3* (Rn00581754-m1), and HCO3^−^/^−^ (Rn00709709-m1). We quantified mitochondrial ribosomal RNA *RPL19* (Rn00821265-g1) as a control gene cDNA. The fold induction was calculated using the comparative 2^−Δ*Ct*^ method. Results were obtained using the 7000 system software, version 1.2.3 (Applied Biosytems).

### Statistical analysis

All statistical analysis for DNA sequences were performed using R. Principal component analyses (PCA) were computed and statistically assessed using a Monte Carlo rank test. The Wilcoxon test was applied to assess statistical significance in bacterial composition between the different samples.

All values are expressed as the mean ± *S.E.M*. Non-parametric tests were used: The Kruskal-Wallis test followed by Dunn's adjusted multiple comparisons to compare more than two groups or Spearman test to correlate two parameters were performed using GraphPad Prism version 5.0 for Windows (GraphPad Software, San Diego, CA, USA). A value of *P* < 0.05 was considered to be statistically significant.

## Results

### SBS patient clinical characteristics and description of the SBS microbiota

**Figure 1:**
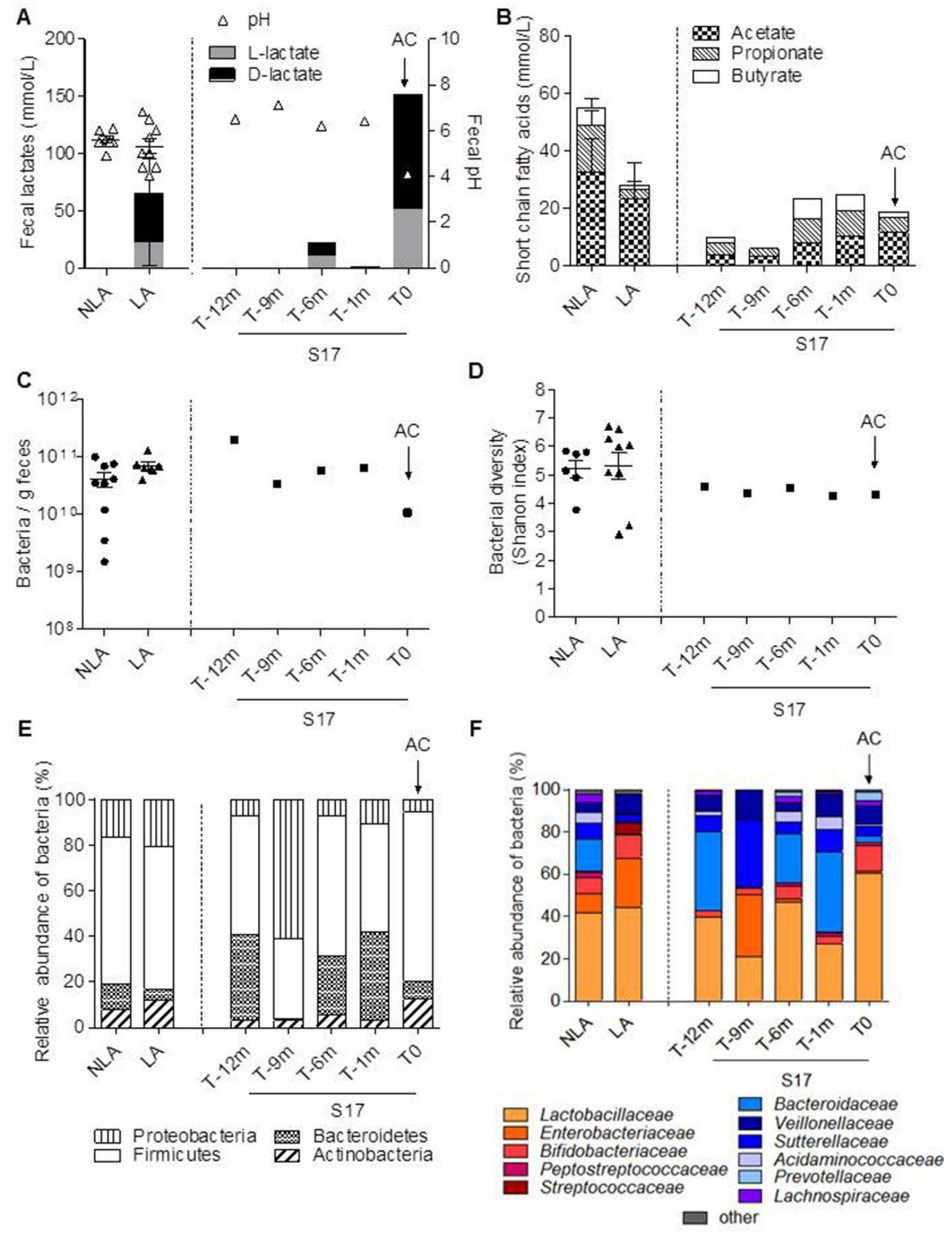
**Fecal fermentative activity and microbial composition of 16 SBS patients clustered in NLA and LA sub-groups and patient S17**. Sixteen patients, individually described in Mayeur et al. (2013), were clustered depending on the presence (LA) or absence (NLA) of lactates in their feces. One supplementary patient (S17) was followed during 1 year because it was known to be at risk for D-lactic acidosis. Samplings of S17 were performed 12 (T-12m), 9 (T-9m), 6 (T-6m), and 1 (T-1m) month before the acidosis crisis (AC at T0). **(A)** Fecal pH and the level of fecal D and L lactates (mmol/L), **(B)** the amount of fecal short chain fatty acids: acetate, propionate, butyrate (mmol/L), **(C)** fecal bacteria per gram, **(D)** fecal microbiota diversity (Shannon index), **(E)** distribution of principal bacterial phyla, **(F)** relative abundance of bacterial families in the feces. Lactate-producing bacteria are indicated in red and orange. Lactate-consuming bacteria are indicated by the different shades of blue.

Seventeen patients with jejuno-colonic anastomosis were enrolled, amongst whom 16 (S1–S16) were sampled once and one (S17) was followed during 1 year with five samplings (Table 1). The clinical and nutritional data of each S1–S16 patient were described in Mayeur et al. (2013). Among S1–S16, seven had no lactate in their feces and were classified as NLA and nine had lactate in their feces and were classified as LA (Table 1 and Figure 1A). We have the opportunity to measure fasting plasma GLP-1 levels in only three patients of the cohort: they were 1.92 (NLA group); 8.54 (NLA group), and 33.3 pmol/L (LA group). Two patients, belonging either to LA or NLA, displayed higher GLP-1 than normal levels of healthy subjects (1.4–5.1 pmo/L; Jeppesen et al., 2000; Gillard et al., 2016).

Patient S17 was followed for 1 year, during which time he was hospitalized once for a D-lactic acidosis crisis at the end of the period (AC; T0; Table 1). During the AC, S17 had a reduced level of plasma bicarbonate (HCO3^−^ from 27 to 22 mmol/L, Table 1) and accumulated fecal lactate with a D/L lactate ratio of >2 (Figure 1A). S17 had a fecal pH that was similar to that of the other SBS patients (NLA or LA) but it dropped to 4.1 during the AC (T0; Figure 1A).

The relative proportion of SCFA in the fecal samples of the NLA and LA sub-groups was acetate > propionate > butyrate. LA and NLA fecal samples had similar levels of acetate and butyrate (Figure 1B), but samples from the LA subgroup had lower propionate levels (3.5 ± 2.8 mmol/L) than those of the NLA sub-group (13.5 ± 6.8 mmol/L, *P* < 0.05). The total fecal SCFA concentration of both the LA subgroup and S17 (all samples) was lower than that of the NLA subgroup (Figure 1B). The level of fecal acetate was lower in S17 than in all other patients. The difference in fecal SCFA concentrations between the LA and NLA subgroups and patient S17 was not related to differences in total bacterial load or diversity (Figures 1C,D and Supplemental Figure 1).

The microbiota composition at the phylum (Figure 1E) and family (Figure 1F) levels was further analyzed by 16S rRNA gene sequencing. *Firmicutes* was the most highly represented phylum comprising 65 and 63% of total bacteria in the NLA and LA sub-groups, respectively (Figure 1E). *Proteobacteria* was the second most dominant phylum (16% in the NLA sub-group and 21% in the LA sub-group) followed by *Bacteroidetes* (11% in the NLA sub-group and 5% in LA the sub-group), and last by *Actinobacteria* (8% in the NLA sub-group and 12% in the LA sub-group). *Firmicutes* was also the most abundant phylum in S17 except at T-9 (Figure 1E). The microbiota of all patients was dominated by the *Lactobacillaceae* (Figure 1F) in accordance with previous results (Kaneko et al., 1997; Mayeur et al., 2013). In the LA sub-group, the lactate-producing bacteria (*Lactobacillaceae, Enterobacteriaceae*, and *Bifidobacteriaceae*) were dominant (>80%), whereas the lactate-consuming bacteria (*Veillonellaceae, Bacteroidaceae, Sutterellaceae*, and *Acidaminococcacea*) were under-represented (<20%). In NLA patients the lactate-consuming bacteria were dominant (40%). Thus, the LA/NLA classification based on the fecal accumulation of lactates reflects the relative abundance of lactate producing- and lactate-consuming bacteria.

The relative abundance of lactate consuming bacteria in S17 was similar to or higher than that in the NLA subgroup except at T0, during the AC. The lactate producing groups (>70%, *Lactobacillaceae* and *Bifidobacteriaceae*) became dominant during the AC (Figure 1F). Overall, in patient S17, the crisis was concomitant with the accumulation of lactate (with a predominance of the D-isoform), a high level of lactate-producing bacteria in feces, and a low level of plasma bicarbonate.

### Fecal transfer from humans to recipient germ free rats

**Figure 2:**
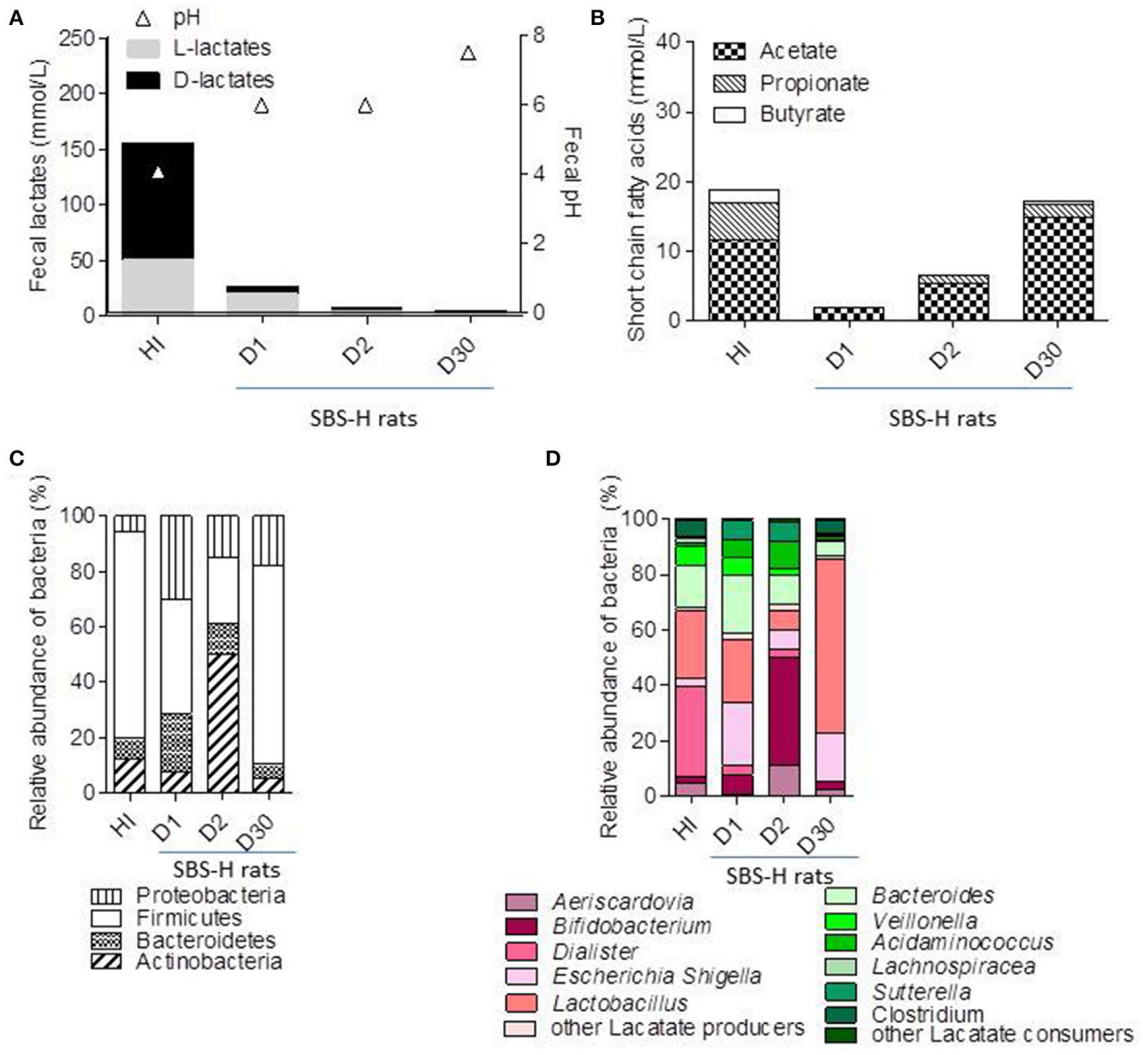
**Fecal fermentative activity and microbiota composition of SBS-humanized rats from 1 to 30 days after fecal transfer**. Feces of S17 patient was recovered at the time of acidosis and used as inoculum (HI) for a fecal transfer in recipient germ free rats (SBS-H). The microbiota was analysis in HI and in fecal samples of SBS-H rats 1 (D1), 2 (D2), and 30 (D30) days after fecal transfer. **(A)** Fecal pH and concentration of fecal D and L lactates (mmol/L) in HI and SBS-H rats (*n* = 6). **(B)** Amount of acetate, propionate, and butyrate (mmol/L) in the HI and in the feces of SBS-H rats. **(C)** Principal bacterial phyla in the HI and the feces of SBS-H rats. **(D)** Relative abundance of bacterial genera in the HI and the feces of SBS-H rats 1 (D1), 2 (D2), and 30 (D30) days inoculation. Lactate-producing bacteria are indicated in different shades of pink. Lactate-consuming bacteria are indicated by the different shades of green.

**Figure 3:**
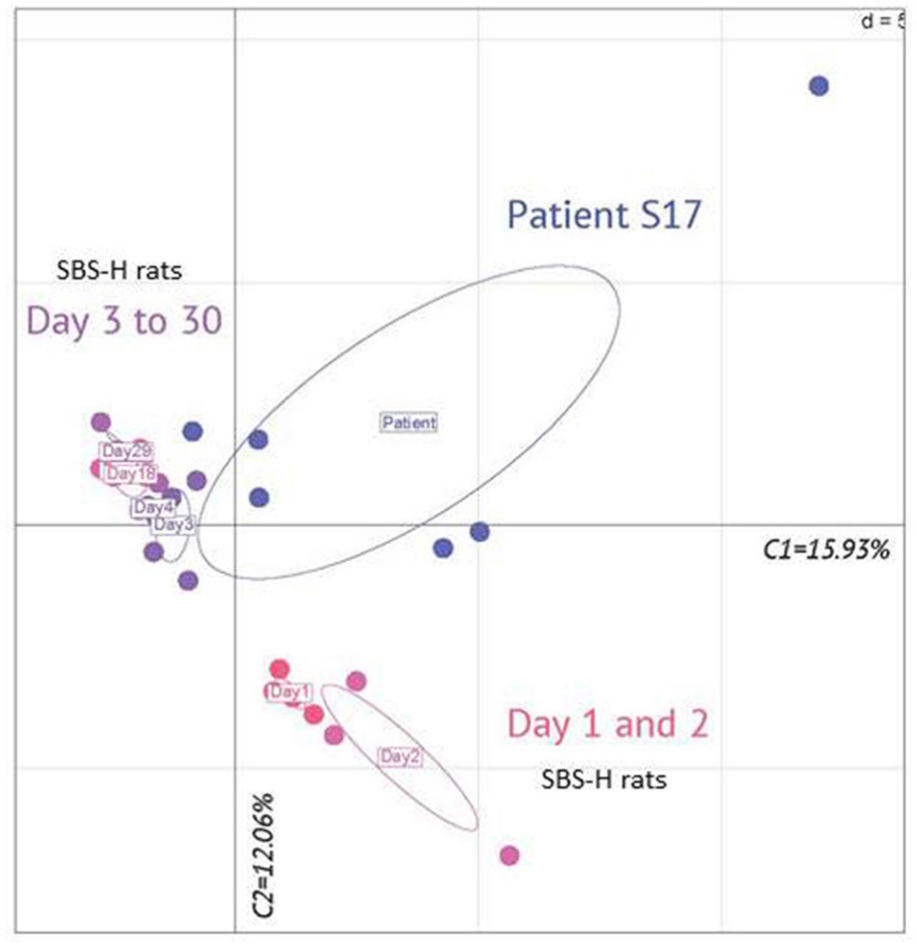
**Principal component analysis of fecal microbiota genera in patient S17 and humanized SBS (SBS-H) rats from 1 to 30 days after fecal transfer**. Principal component analysis of fecal microbiota genera from data obtained by pyrosequencing. The microbiota of SBS-H rats of D1 (*n* = 4) and D2 (*n* = 3) clustered together. The microbiota of SBS-H rats of D3 (*n* = 5), D4 (*n* = 3), D18 (*n* = 4), and D29 (*n* = 4) clustered together. Each point for patient S17 represents samplings during 1 year [T-12 (in duplicate), T-9, T-6, T-1, and T0]. T-9 is the most distant point. Component 1 explains 15.93% of the variance; Component 2 explains 12.06% of the variance.

The fecal sample of S17, at the time of the AC (T0), was prepared and diluted in an anaerobic chamber as inoculum (HI) and transferred by oral gavage into recipient germ free (GF) rats. We assessed the fermentative activity and microbial composition in rats harboring an SBS-derived microbiota (SBS-H) 1 (D1), 2 (D2), and 30 days (D30) after gavage (Figures 2, 3). The amount of total bacteria reached 10^9^–10^10^ bacteria/g of feces, similar to the load of the inoculum (10^10^), indicating proper colonization of the GF rats by the SBS patient microbiota (Supplemental Figure 2). By D30, the fecal pH increased from 4 (at HI) to 7.5, whereas total fecal lactates decreased from 150 mmol/L (at HI) to 2 mmol/L (Figure 2A). The D-lactate accumulation in feces and D-encephalopathy observed in donor (S17) were not reproduced in SBS-H rats. We then tested whether D-lactate acidosis could be provoked after a challenge with lactose. The addition of lactose in the drinking water for 5 days did not lead to any further accumulation of lactate in the feces in SBS-H rats. Our results suggest that the accumulation of lactate observed in S17 patient with a resection but was not recapitulated after a fecal transfer in rats with intact gut. After transfer, the total amount of lactates and butyrate diminished, while the amount of acetate and propionate increased in the SBS-H fecal samples. Thirty days after transfer, the fermentative profile in the SBS-H rats tended to be close to fermentative activity of inoculum (HI; Figure 2B).

The Figures 2, 3 illustrate the progressive adaptation of microbiota (sampling in S17 patient) after the transfer into recipient germ free rats. During the 2 first days following the fecal transfer (D1 and D2), the microbiota is adapting to its new environment (from a resected gut in human to an intact gut in recipient rats). Thus, the microbiota ecosystem changed profoundly during the 2 days following its arrival into a new digestive environment, reflecting an adaptive phase, whereas the composition of the SBS-H microbiota seemed to stabilize from D3 to D30 (Figure 3). All measured parameters (fecal lactates, SCFA levels, microbiota composition, pH-value) did not change between D3 and D30 (data not shown). The Figure 3 also indicates that the microbiota, from D3 to D30 days was closer to the inoculum than was the microbiota during the 2 first days after transfer. The main phylum represented in the HI was *Firmicutes* (74%), followed by *Bacteroidetes* (15%), *Actinobacteria* (7%), and *Proteobacteria* (3%) and at D30 after the fecal transfer, the main represented phylum remained *Firmicutes* (71%), followed by the *Proteobacteria* (18%), *Bacteroidetes* (5%), and *Actinobacteria* (5%; Figure 2C). Thirty days after transfer, the SBS-H microbiota conserved characteristics of the SBS-microbiota, predominated by lactate-producing bacteria (mainly *Lactobacillus* and *Bifidobacterium*), a low level of lactate-consuming bacteria (*Bacteroides* and *Veillonella*), and undetectable *C. leptum* (Figure 2D).

### The trophic effect of the SBS-microbiota on the colon epithelium in SBS-H rats

**Figure 4:**
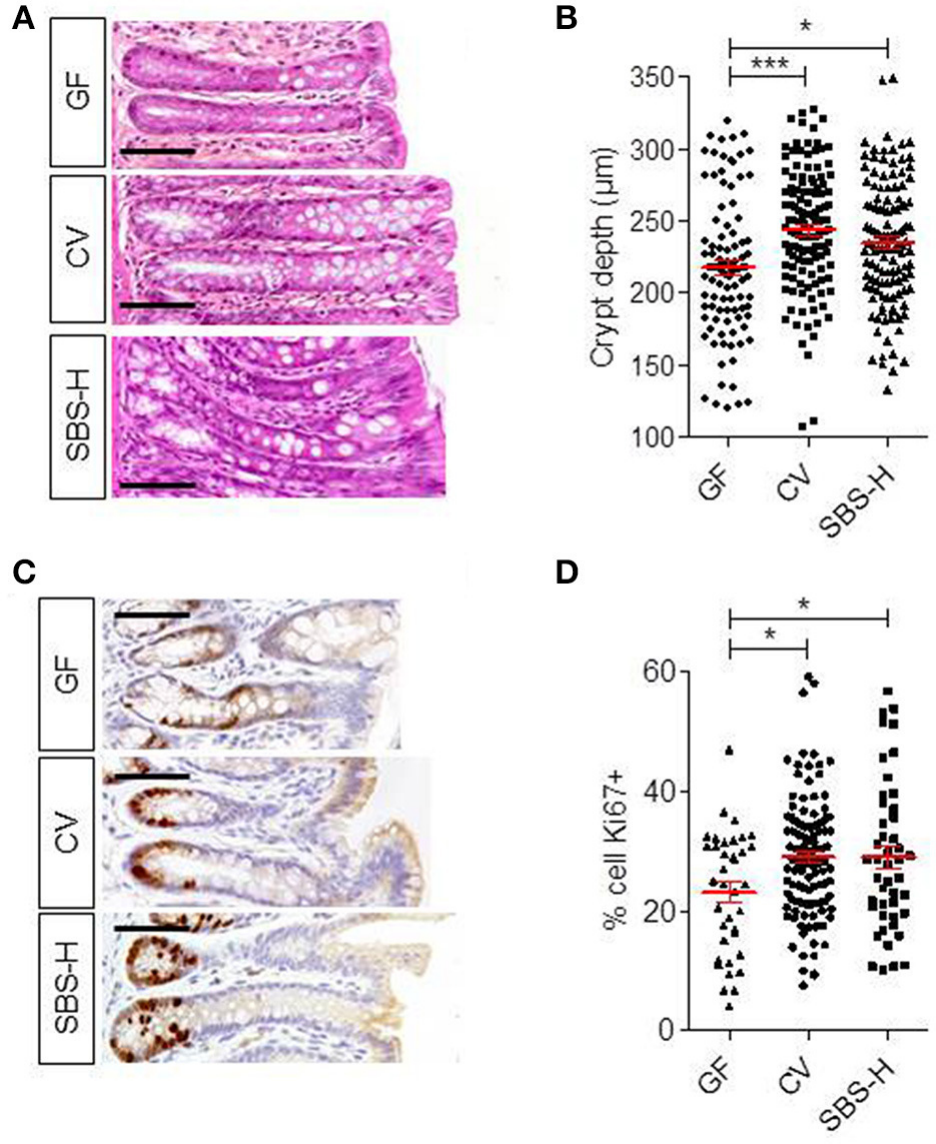
**Epithelial structure and proliferation in GF, CV, and SBS-H rats. (A)** Representative image of a colon section from GF, CV, and SBS-H (D30) rats. Scale bar, 50 μm. **(B)** Crypt depth (μm) in the colon of GF (*n* = 6), CV (*n* = 4), and SBS-H rats (*n* = 6). Twelve crypts in one histologic section were measured for each rat. Each dot represents one measurement. Data are expressed as the mean ± SEM, ^*^p < 0.05, ^***^p < 0.001 based on the Kruskal-Wallis test followed by Dunn's adjusted multiple comparisons to compare the three groups. **(C)** Representative image of Ki67 positive cells in colon sections from GF, CV, and SBS-H rats. Scale bar, 50 μm. **(D)** Ki67 positive cells expressed as the percentage of total cell number in colon crypts from GF, CV, and SBS-H rats. Ten crypts per rat were analyzed. Data are expressed as the mean ± SEM, ^*^p < 0.05 based on the Kruskal-Wallis test followed by Dunn's adjusted multiple comparisons to compare the three groups.

SBS patients usually display deeper crypts than healthy controls (Joly et al., 2009). We thus compared the colonic crypt depth of SBS-H rats (at D30) to conventional (CV) rats (Figures 4A,B). Histological analysis showed that there was no difference in the crypt depths between CV (244 ± 32 μm) and SBS-H (235 ± 37 μm) rats. Crypt depths were less in GF rats (217 ± 38 μm) than in CV (*P* < 0.05) rats, as described previously (Cherbuy et al., 2010). There were a higher number of Ki67-positive cells per crypt both in CV and SBS-H rats than in GF rats (Figures 4C,D, *p* < 0.05). We thus observed a morphogenic effect triggered by two different microbial communities: the SBS-microbiota and the CV microbiota.

### Effect of the SBS-derived microbiota on colonic transporter expression

**Figure 5:**
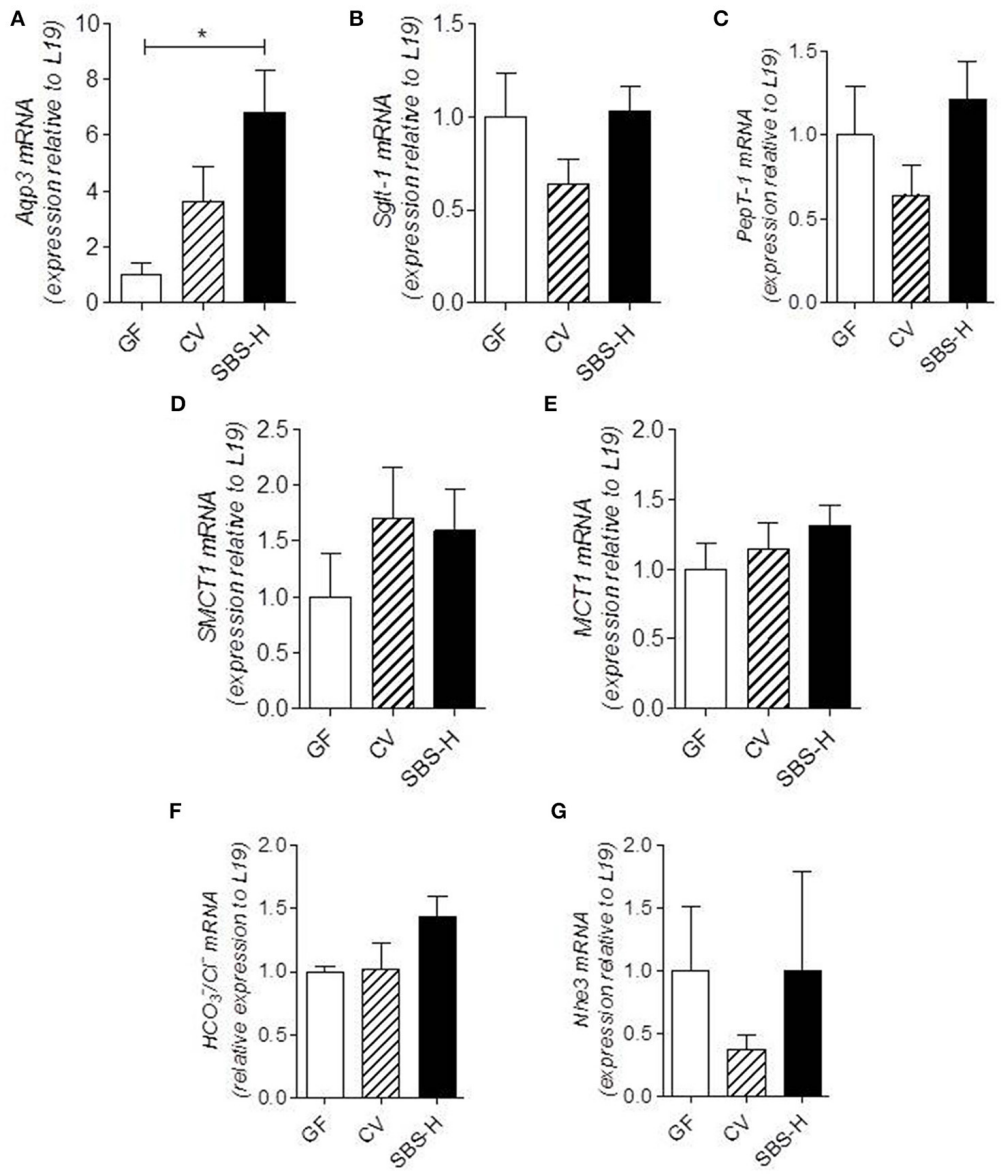
**Transporter expression in colon mucosa of GF, CV, and SBS-H rats**. mRNA levels of Aqp3, Sglt-1, PepT-1, SMCT-1, MCT-1, ${\text{HCO}}_{3}^{-}$/Cl^−^, and Nhe3 normalized to the L19 housekeeping gene in colon mucosa from GF (*n* = 4), CV (*n* = 4), and SBS-H rats (*n* = 4) **(A–G)**. Data are expressed as the mean ± SEM, ^*^P < 0.05 based on the Kruskal-Wallis test followed by Dunn's adjusted multiple comparisons to compare the three groups.

We explored how SBS-microbiota may change the absorptive capacities of the colon by examining the expression of different nutrient and solute transporters that play a key role in nutrient and water salvage. There tended to be a higher level of mRNA encoding *Aqp3*, an aquaporin water channel, in the colon of SBS-H rats than CV rats and it was statistically higher (*p* < 0.05) in GF rats (Figure 5A). The mRNA levels of *SGLT-1, PepT1, SMCT-1, MCT-1, HCO3*^−^*/Cl*^−^, and *Nhe3* transporters in SBS-H rats were not statistically different from those in CV and GF rats (Figures 5B–G). The SBS-derived microbiota thus promoted higher expression of *Aqp3* mRNA in the colon than that observed in GF rats.

### The effect of the SBS-derived microbiota on endocrine functions

**Figure 6:**
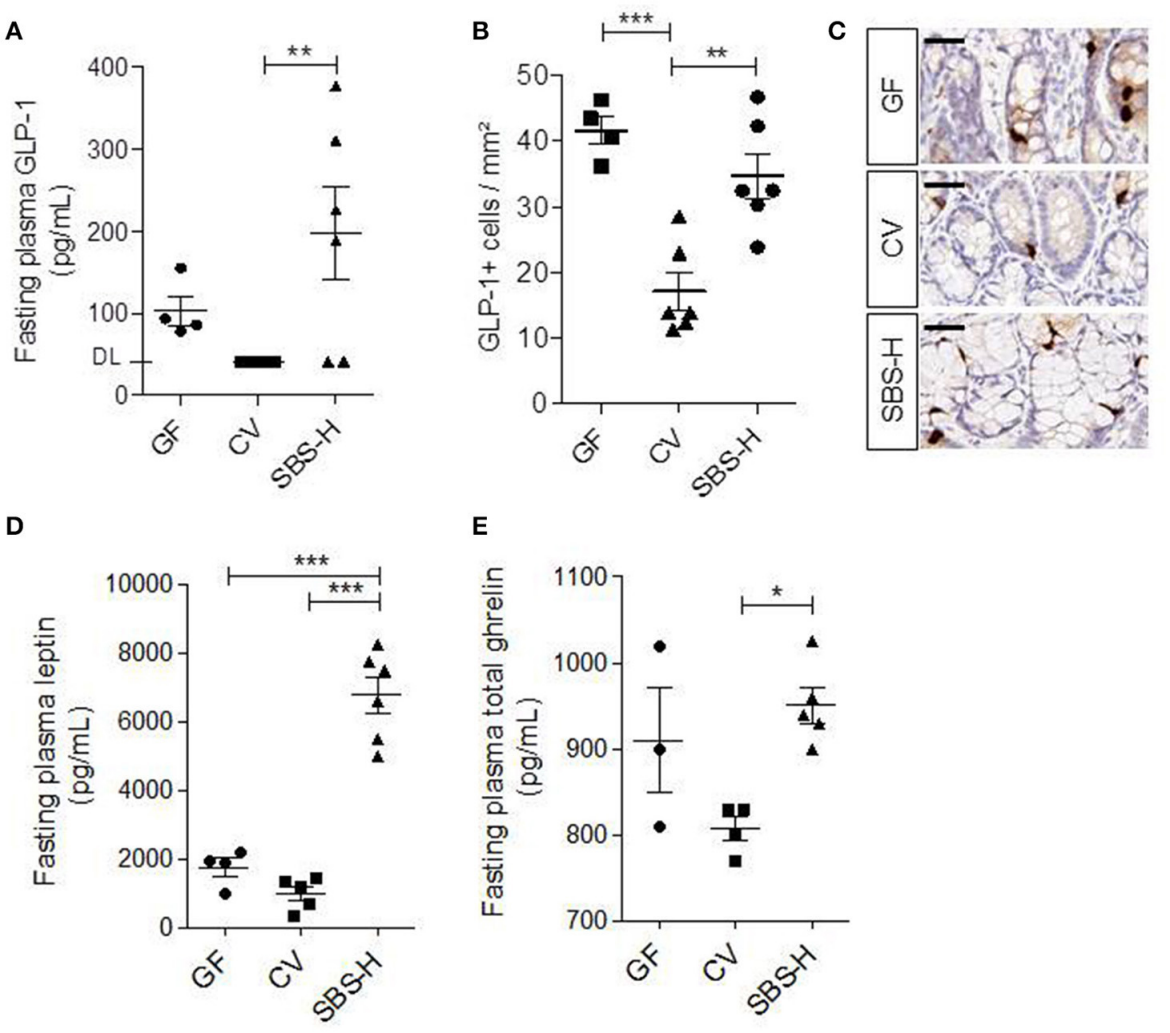
**GLP-1, leptin and ghrelin in GF, CV and SBS-H. (A)** Fasting plasma levels of GLP-1 in GF (*n* = 4), CV (*n* = 5), and SBS-H (*n* = 6) rats. Plasma GLP-1 levels of all CV rats and 2 SBS-H rats were below the limit of detection. **(B)** Number of GLP-1 positive cells per mm^2^ in GF, CV, and SBS-H rats (one section per rat). **(C)** Representative image of GLP-1 immunostaining (brown cytoplasm), of colon mucosa sections from GF, CV, and SBS-H rats. Scale bar, 50 μm. **(D)** Fasting plasma levels of leptin (pg/mL) in GF (*n* = 4), CV (*n* = 5), and SBS-H rats (*n* = 6). **(E)** Fasting plasma levels of ghrelin (pg/mL) in GF (*n* = 3), CV (*n* = 4), and SBS-H rats (*n* = 5). Data are expressed as the mean ± SEM, ^*^p < 0.05, ^**^p < 0.01, ^***^p < 0.001 based on the Kruskal-Wallis test followed.

We measured the plasma levels of GLP-1, leptin, and ghrelin in SBS-H, CV, and GF rats (Figure 6). The level of GLP-1 was not statistically different between SBS-H and GF, even if it tended to be higher in some SBS-H rats (Figure 6A). However, SBS-H rats had higher plasma levels of GLP-1, more L cells and higher concentration of plasma ghrelin than CV rats (Figures 6A,B,D). The location and global morphology of L cells were similar for all rats (Figure 6C). The high level of GLP-1 observed in SBS-H rats was in concordance with the high level of GLP-1 described in SBS patients (Jeppesen et al., 2000). We observed a significant four and 7-fold increase in the level of fasting plasma leptin in SBS-H rats relative to both GF and CV rats, respectively (*p* < 0.001, Figure 6D).

## Discussion

The microbiota of SBS is shaped by the reduction of the small intestine disrupting the oro-anal gradient in the digestive tract and in return, intervenes at several levels in the clinical outcomes of patients. The SBS-microbiota is actively involved in accumulation of fecal lactate, susceptibility to D-acidosis and post-resection adaptation.

First, the accumulation of lactate in feces is dictated by the abundance of lactate producing bacteria in microbiota of patients. The classification of SBS patients could be used routinely by clinicians to distinguish patients who never accumulate lactate from those at risk to develop an acidosis crisis. We confirm that the accumulation of fecal D-lactate and the drop in the level of plasma bicarbonate are two parameters associated with the D-lactic acid encephalopathy crisis by following one patient over 1 year. The fecal human material at the time of the encephalopathy was transferred into recipient germ free rats (SBS-H rats). Even if, the SBS-H microbiota conserved some characteristics of the inoculum, lactate did not accumulate and the D-encephalopathy was not reproduced in SBS-H rats. The SBS-microbiota is a causative agent of lactate accumulation, that can cause a D-lactate acidosis in the context of reduced small bowel length.

Second, the SBS-microbiota seems also to be a reservoir of multiple and complex signals that could modify the post-resection adaptation. Our study describes that fecal transfer of SBS-associated microbiota into recipient germ-free rats triggers modifications of colon by crypt deepening but does not reduce the level of GLP-1 and ghrelin of GF. Thus, SBS-H and GF rats have higher amount of these hormones than rats housing a conventional microbiota. The SBS-microbiota may favor energy recovery since its transfer into GF is accompanied by high level of plasma leptin.

Our cohort of 16 SBS patients is representative of SBS with jejuno-colonic anastomosis associated with severe malabsorption and hyperphagia (Mayeur et al., 2013). Patients in whom lactate accumulated (LA), known to be at risk of developing an acidosis crisis, had a gut microbiota enriched in lactate-producing bacteria (*Lactobacillaceae, Bifidobacteriaceae*, and *Enterobacteriaceae*) with a lower proportion of lactate-consuming bacteria (*Veillonellaceae, Bacteroidaceae)*. Thus, the classification of two groups (LA and NLA) reflects the relative abundance of lactate-producing and lactate-consuming bacteria. The specific composition and fermentative activity of the microbiota from SBS patients result from the luminal constraints due to the extended resection of the small intestine.

The fecal transfer illustrates the adaptability of a specific ecosystem in its environment. The inoculum coming from a truncated intestine was transferred into a complete digestive tract. The inoculum adapted progressively to its new gut environment in recipient GF rats during the first 2 days and then stabilized 3 days after fecal transfer. On day 30, the composition of the microbiota established in the gut of SBS-H rats was relatively similar to the human fecal microbiota used as inoculum, as has been reported in previous studies (Chung et al., 2012; Crouzet et al., 2013). Three main characteristics of the SBS-derived microbiota were conserved: the dominance of *Lactobacillus* and the absence of *C. leptum* and butyrate. We did not observe an accumulation of lactate in the feces of SBS-H rats (even after supplementing the drinking water with lactose), suggesting that the intact small bowel of the recipient rats protected them from lactate accumulation. Thus, the reduction of the small intestine appears to be required for the accumulation of lactate but is not sufficient, as fecal lactate is only observed in certain sub-groups of patients.

Intestinal failure includes the malabsorption of vitamins, trace elements, electrolytes, water, and energy. The absorptive function of the colon mucosa increases over time in SBS patients with colon in continuity, despite residual malabsorption, thus decreasing their need for PN (Nordgaard et al., 1994, 1996). Patients with preserved colonic function gain more energy, especially by fermenting carbohydrates (Nordgaard et al., 1996). Several published studies suggest that the expression of some transporters may exhibit adaptive flexibility and over expression in SBS. However, there is no consensus and it is not established whether, which and where precisely in the gut transporters are boosted after resection (Mayeur et al., 2016). The increase in the level of Aqp3 has been described in a pre-clinical model in resected rats (Tsujikawa et al., 2003) as we observed in SBS-H rats. It could be speculate that the increased Aqp3 may help to save water and exchange metabolites in SBS-H rats; the mechanisms underlying this over-expression are not known. We have previously described both in Humans and in preclinical models a gut epithelium restructuring consequently to the resection. With the fecal transfer, we wanted to know if the SBS-microbiota sent specific signals leading to deeper absorptive surface. The SBS-derived microbiota after fecal transfer in SBS-H rats, triggers crypt deepening and increases proliferation in the colon but this was also observed in CV rats housing a different microbiota. This result is concordant with our previous results showing that different types of microbe communities (either a microbial population rich in primo-colonizing bacteria or a population representative of adult microbiota) have similar morphogenic effect on colon epithelium (Tomas et al., 2013). The trophic effect was not recapitulated when GF rats were mono-colonized with one single commensal, suggesting that diversity of the inoculum is required to send signals that shape the epithelial mucosa (Rul et al., 2011; Turpin et al., 2013; Wrzosek et al., 2013; Tomas et al., 2015; Hoffmann et al., 2016). It may now be instructive to test whether the microbiota coming from SBS patients that are in demand of energy because of their malabsorption have greater morphogenic potential than microbiota coming from subjects with less malabsorption.

Several studies describe an increase in fasting plasma GLP-1 levels in SBS patients with a jejuno-colonic anastomosis or in resected rats (Jeppesen et al., 2000; Gillard et al., 2016). In the present work, in two patients of the cohort in whom it was measured, the plasma GLP-1 concentration was indeed higher than normal levels. We also observed that SBS-H rats had higher plasma GLP-1 levels associated with a higher number of L cells than CV rats. GLP-1 is a mediator of the colonic-ileal brake (inhibits gastro-intestinal motility) in response to nutrients (Xiao et al., 1999; Van Citters and Lin, 2006). Recently, Wichmann et al., reported that GF mice and antibiotic-treated mice, with reduced SCFA levels, have higher basal plasma GLP-1 levels than CV mice (Wichmann et al., 2013). Colonic L cells may sense the need of energy and regulate basal GLP-1 secretion to compensate the absence of microbiota in GF (Zietek and Rath, 2016). The increase of fasting plasma GLP-1 levels both in GF and in SBS-H rats may be an adaptive response to high demand of energy that slows down intestinal transit and consequently allows greater nutrient absorption. So, microbiota, through the short chain fatty acids production is a key actor in triggering GLP-1 secretion (Zietek and Rath, 2016). The SCFA content is very different between CV, GF, and SBS-H rats, with no butyrate in the SBS-H rats and GF, whereas the butyrate concentration was up to10 mM in CV rats (Tomas et al., 2013) and data not shown). The increased level of GLP-1 (in comparison with CV) may also be linked to the richness of lactic acid bacteria in the SBS-microbiota (Okubo et al., 2015). Nevertheless, the GLP-1 level seems to be independent of lactate overload since it was observed in SBS-H rats and in some patients that did not accumulate lactate. The higher level of GLP1 in the presence of the SBS-microbiota may favor energy recovery. A study evaluated the effect of a GLP1 agonist (Exenatide) in five SBS patients with ≤ 90 cm of small bowel and clinical evidence of nutritional deprivation. All five patients had immediate improvement, including bowel movements, and parenteral nutrition was successfully halted in three patients (Kunkel et al., 2011). It may be instructive to characterize which bacteria are involved in stimulating GLP-1 production by the host.

We have recently demonstrated that the level of ghrelin, the unique orexigenic gut hormone, was elevated in rat models of SBS and in patients with jejuno-colonic anastomosis without changes in leptin levels (Gillard et al., 2016). Here, we observed increased levels of both ghrelin and leptin in SBS-H rats housing an SBS-microbiota. In patients, the high levels of GLP-1 and ghrelin are not accompanied by increased leptin levels, probably due to their truncated gut and severe malabsorption. The deeper crypts, the high levels of GLP-1 and ghrelin, the increase in Aqp transporter could account for higher efficiency to salvage energy in SBS-H rats. Since SBS-H rats had no resection, this high ability in recovering energy is coherent with their high level of leptin. It is likely that a microbiota coming from an organism that needs energy triggers signals that may contribute to energy recovery, as has been proposed after exposure to cold (Chevalier et al., 2015). Based on fecal transfer experiments, we propose that the SBS-microbiota carries signals that contribute to the morphological and functional adaptations described in the remnant colon of patients. But, we cannot exclude that enhanced GLP-1, ghrelin, and leptin over-expression also alters microbiota.

The fecal transfer has been widely employed in many studies to highlight causal relationships between gut microbiota and clinical characteristics of various diseases (Nguyen et al., 2015). The critical end point of this method is the selection of the best-reliable control. We chose to compare SBS-H rats with GF and CV rats, receiving their mother-derived microbiota at birth by vertical transmission. Although the use of CV and GF rats may lead to bias, it represents a first step toward a better understanding of the SBS-microbiota functions and signals. It may be informative to transfer a microbiota coming from different sub-groups (such as NLA or LA) or from patients suffering from other malabsorptive syndromes after different types of surgeries.

There is a close relationship between the specific composition of SBS microbiota and post-surgery outcome and adaptation. Our study may help to design nutritional supply or future fecal transfer strategies to avoid fecal lactate accumulation, prevent D-acidosis crisis, improve adaptation and decrease need of PN. SBS illustrates how microbial metabolites, such as D-lactate, can alter neurological functions highlighting the gut/brain dialogue and we also confirm the link between hormones secretion and microbiota.

## Author contributions

LG, CM, PL, MT, and FJ initiated and designed the study, obtained, analyzed and interpreted the data, wrote the manuscript. LG, IP, and FJ recruited the patients, obtained, and compiled the clinical data. PL analyzed all sequencing data. VR contributed to fecal transfer. JL, AB, PL have critically read the manuscript.

## Funding

This research was supported by the Institut National de la Recherche Agronomique (INRA) and by a grant from Biocodex through the Société Nationale Française de Gastro-entérologie. LG received a Ph.D. grant from the French Ministry of Education and Research.

### Conflict of interest statement

The authors declare that the research was conducted in the absence of any commercial or financial relationships that could be construed as a potential conflict of interest.

## Abbreviations

SBS: short bowel syndrome
SCFAs: short chain fatty acids
Aqp-3: aquaporin 3
SGLT-1: Sodium-glucose co-transporter 1
PEPT1: proton-coupled oligopeptide transporter
MCT1: monocarboxylate transporter 1
SMCT1: sodium-coupled monocarboxylate transporter 1
NHE3: Na(+/) H(+) exchanger 3.
