# Supplementary material for: Microbiota Is Involved in Post-resection Adaptation in Humans with Short Bowel Syndrome
Source: Front Physiol. 2017 Apr 19;8:224. doi: 10.3389/fphys.2017.00224 (PMC5395573; doi:10.3389/fphys.2017.00224)

Supplemental figure 1: Fecal microbiota diversity (Simpson index) of NLA and LA sub-groups and patient S17.

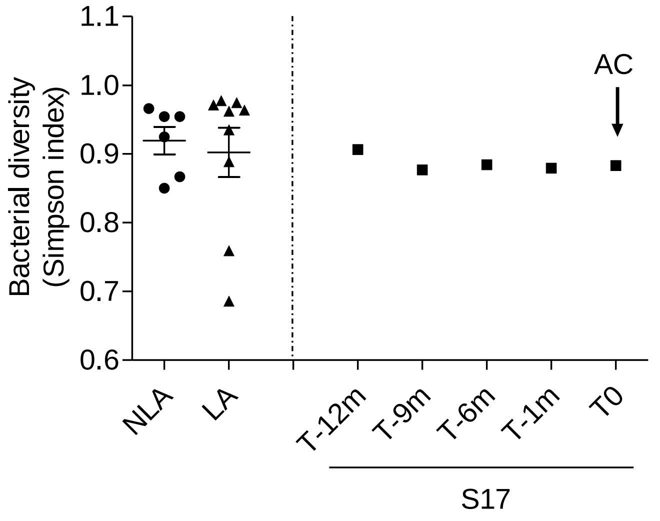

Supplemental figure 2: Total bacteria per gram of feces in S17-inoculum and in SBS-H feces 1, 2, and 30 days after fecal transfer. The amount of total bacteria was obtained by quantitative PCR.

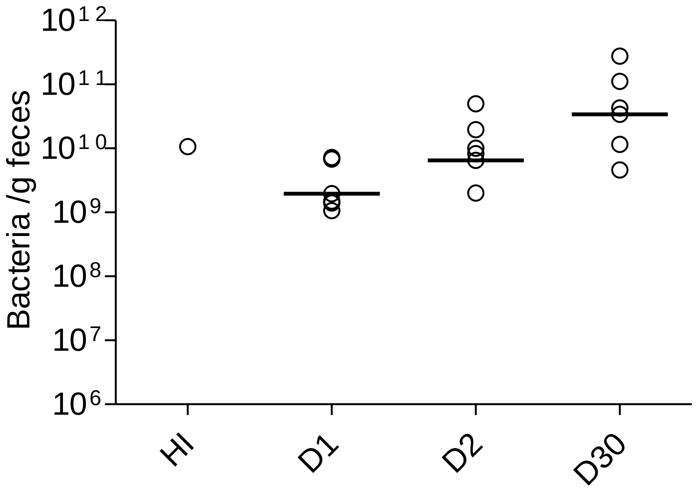

Supplement: Supplementary file 1 [file DataSheet1.pdf]
